# Preclinical approaches to studying liver and kidney fibrosis: models and methodologies

**DOI:** 10.3389/fphar.2026.1718048

**Published:** 2026-02-16

**Authors:** Abhilasha Tiwari, Shivam Singh, Rajesh Kumar Sharma, Sandeep Kumar

**Affiliations:** 1 Department of Pharmacology, Nims Institute of Pharmacy, Nims University Rajasthan, Jaipur, India; 2 Department of Pharmaceutics, Nims Institute of Pharmacy, Nims University Rajasthan, Jaipur, India; 3 Department of Pharmacy, Faculty of Health and Allied Science, KAAF University, Accra, Ghana; 4 Chitkara College of Pharmacy, Chitkara University, Rajpura, Punjab, India

**Keywords:** chronic kidney disease, extra cellular matrix, hepatic stellate cell, liver fibrosis, renal fibrosis

## Abstract

Organ fibrosis, notably affecting the liver and kidneys, remains a major contributor to global morbidity. This review examines the pathophysiology, molecular mechanisms, and preclinical models used to study hepatic and renal fibrosis. In liver fibrosis, hepatic stellate cell activation, chronic inflammation, and extracellular matrix accumulation are central features, while renal fibrosis involves myofibroblast activation and redox-mediated signaling pathways. The present review highlights both *in vitro* and *in vivo* models such as the carbon tetrachloride, bile duct ligation, and dimethylnitrosamine-induced liver fibrosis models, as well as renal fibrosis models like unilateral ureteral obstruction (UUO), subtotal nephrectomy, and adriamycin nephropathy. It also emphasizes advanced experimental platforms including liver slice systems and stem cell transplantation techniques. All these above-mentioned models of hepatic and renal fibrosis involve immune cells directly or indirectly, e.g., cytokines, chemokines, and growth-promoting factors in renal fibrosis UUO model. By integrating molecular insights and experimental techniques, this review provides a comprehensive guide for future therapeutic strategies aimed at mitigating fibrosis in chronic liver and kidney diseases.

## 1 Introduction

In organ fibrosis, a medical condition brought on by repeated or persistent tissue injury, extracellular matrix (ECM) proteins, particularly collagens, build up excessively and persistently. In this aberrant and dysregulated wound-healing process, activated fibroblasts and myofibroblasts replace normal parenchymal tissue with fibrotic scar tissue, which ultimately results in morphological distortion and growing functional impairment of the injured organ.

When the liver experiences chronic inflammation or repeated injury, there is an excessive build-up of scar tissue, which leads to liver fibrosis. Fibrosis can eventually result from the majority of chronic liver diseases. Scar tissue cells are functionally incapable of self-repairing or regenerating like healthy liver cells. Fibrosis can therefore lower total liver function and hinder the organ’s capacity to regenerate. The blood flow within the liver can also be obstructed or restricted by fibrotic scar tissue. This may cause healthy liver cells to starve to death and eventually produce additional scar tissue. Numerous cellular pathways are implicated in the mechanism of fibrosis; however, hepatic stellate cells (HSC) seem to be the main cell type involved. Liver fibrosis is the result of chronic hepatic damage brought on by a number of liver illnesses, such as fatty liver disease, hepatitis B and C, and others. In developed nations, the main contributors to liver fibrosis include excessive alcohol consumption and infections with hepatitis B (HBV) and hepatitis C (HCV) viruses, as well as metabolic disorders linked to obesity, insulin resistance, and diabetes (Crespo Yanguas et al., 2016). The primary reasons of the rise in liver disease worldwide include lifestyle factors like MASLD, which affects 38% of people, and increasing ALD, which is linked to poor diets, obesity, and alcohol intake. Despite efforts to prevent new HBV and HCV infections, 1.5 million new instances of chronic viral hepatitis occur annually, causing liver damage. Hepatotoxic drugs, environmental pollutants, rising rates of cirrhosis and liver cancer, and socioeconomic inequality all contribute to the liver disease problem. Liver cancer, viral hepatitis (HBV/HCV), alcohol-related liver disease, and metabolic dysfunction-linked steatotic liver disease are the main causes of the over 1.26 million annual deaths caused by liver disease, which is a major global health concern (Gan et al., 2025). There are two main blood vessels that supply the liver the hepatic artery and the portal vein. “The portal vein carries venous blood from the intestines and spleen to liver*”.* From the celiac axis, the hepatic artery delivers arterial blood to the liver. The Glisson’s capsule, which is mostly made of connective tissue, encloses the liver (Acharya et al., 2021). The liver is placed inside a Glisson’s capsule and is split by connective tissue into polygonal portions known as lobules. Every lobule has a distinct layout that is affected in different ways depending on the stage of liver fibrosis or cirrhosis (Sasse et al., 1992). Because liver function and this arrangement are so closely related, cirrhosis entirely disrupts liver function, which can lead to issues. The glomerulus and the tubulus are the two main components of the nephron, the main kidney unit. Injury to glomerular cells, such as mesangial cells, basement membrane, podocytes, or tubular cells, can start renal fibrosis. Immune complex-mediated glomerulonephritis is caused by systemically produced immune complexes that preferentially deposit on glomerular endothelium and mesangial cells, as well as occasionally along the podocyte-facing (subepithelial) surface, in a variety of clinical situations (Couser, 1990).

Renal fibrosis, characterized by an abnormal accumulation of extracellular matrix proteins in the tubulointerstitial and glomerular compartments, is the last common pathway for most chronic kidney diseases. Globally, the prevalence of chronic kidney disease (CKD) has dramatically increased during the last 30 years. Globally, the number of persons (aged ≥20) with CKD increased from around 378 million in 1990 to an expected 788 million in 2023. This translates to an approximate age-standardized adult prevalence of 14.2%. That year, CKD was the 10th most common cause of death globally, accounting for roughly 1.48 million deaths (Mark et al., 2025). Long-term injury to tubular epithelial cells, podocytes, mesangial cells, and endothelial cells causes oxidative stress, inflammatory cytokines, and significant profibrotic signaling pathways. Extracellular matrix deposition is primarily caused by fibroblast activation and myofibroblast differentiation, both of which are stimulated by the RAAS and transforming growth factor-β1 (TGF-β1)/Smad signaling. Other processes include macrophage-driven inflammation, microvascular rarefaction, and EMT further speed up matrix accumulation. When progressive renal architectural distortion impairs glomerular filtration and oxygen diffusion, renal function is irrevocably lost.

### 1.1 Pathogenesis of liver fibrosis

Liver fibrosis is a slow healing response to chronic hepatic injury. In reaction to hepatocyte apoptosis or necrosis, which releases ROS, DAMPs, and apoptotic bodies, hepatic macrophages (Kupffer cells) secrete pro-fibrotic cytokines such as transforming growth factor-β (TGF-β), (TNF-α), and platelet derived growth factor (PDGF). These mediators cause hepatic stellate cells (HSCs) to transdifferentiate into myofibroblast-like cells that express α-smooth muscle actin. Activated HSCs, which also overproduce collagen types I and III, collagen IV, laminin, and fibronectin, are the main source of ECM in the fibrotic liver. Long-term activation of the TGF-β/SMAD, PDGF, and NF-κB signaling pathways promotes the accumulation of ECM and prevents matrix breakdown, leading to progressive fibrosis, cirrhosis, and architectural deformation.

Mutations in the SERPINA1 gene, which codes for the serine protease inhibitor Alpha-1 antitrypsin (AAT), cause AAT deficiency (OMIM 613490), an autosomal recessive (codominant) disorder. The mostly hepatic cell-synthesised protein prevents lung damage from proteolytic degradation by blocking proinflammatory proteases such as neutrophil elastase.

**FIGURE 1:**
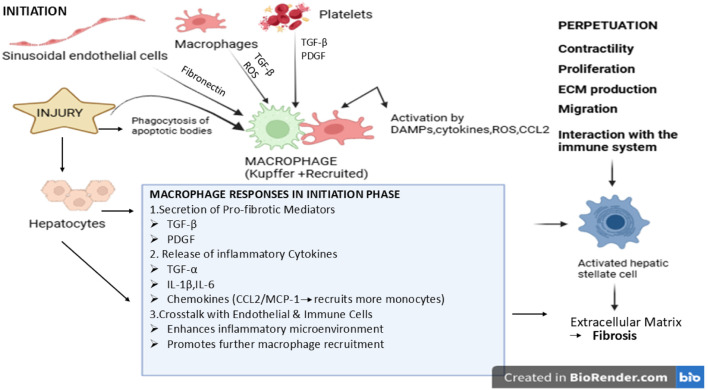
Stellate cell mechanism of action.

Although the incidence of AAT insufficiency is 1 in 2,000–5,000, there are less people with diagnoses than there should be (Scorza et al., 2014). Alcoholic liver disease (ALD) significantly increases morbidity and mortality since it is a primary cause of cirrhosis, liver cancer, and acute and chronic liver failure (Stickel et al., 2017). HSCs, which are quiescent and located in the Disse space (Acharya et al., 2021) of a healthy liver, express glial fibrillary acidic protein (GFAP) and act as stores of retinol esters, a type of vitamin A. One of the main stages of liver fibrosis is the activation of HSCs, which causes these cells to take on characteristics of myofibroblasts (Geerts et al., 1998). There are two main stages to the complex process of activating HSCs: initiation and perpetuation. If the injury heals, the fibrosis will resolve after the initiation step. When lipopolysaccharide is released from the gut following liver damage, the production of paracrine activation, reactive oxygen species (ROS), and apoptotic bodies serve as the initiating triggers (Lee and Friedman, 2011). These triggers increase cell sensitivity, and if they continue, HSCs keep their active state, which encourages the build-up of extracellular matrix (ECM) and long-term inflammation. Activated HSCs and other fibrogenic cells promote the production of ECM during liver fibrogenesis by activating key molecular pathways like TGF-β/SMAD, PDGF, and NF-κB signaling, which upregulate the transcription of collagen I, III, IV, laminin, and fibronectin. The main collagen fibers produced in the extracellular matrix during liver fibrosis are type I and type III collagens, which comprise most of the fibrotic scar, and increased type IV collagen deposition in the basement membrane. Oxidative stress, inflammatory cytokines, and extended ethanol metabolism all promote excessive matrix deposition. Proteolytic enzyme dysregulation also hinders the degradation of the extracellular matrix. Matrix metalloproteinases (MMP-2, MMP-9), which normally break down collagen and other ECM components, are inhibited while their endogenous inhibitors, tissue inhibitors of metalloproteinases (TIMP-1, TIMP-2), are elevated. Fibrosis develops more quickly and ECM turnover is reduced due to this imbalance between MMPs and TIMPs. Therefore, both reduced matrix breakdown and increased ECM synthesis have an impact on scar formation in alcoholic liver disease. In this case, other ECM-producing cells, such as portal fibroblasts, aid in the creation of scars in the liver (Lemoinne et al., 2013). Bone marrow-derived myofibroblasts and epithelial cells that shift from epithelium to mesenchymal as shown in Figure 1 (Crespo Yanguas et al., 2016). However, hepatic macrophages (Kupffer cells) do not transdifferentiate into myofibroblasts. Instead, by generating inflammatory and profibrotic cytokines that activate hepatic stellate cells and promote the production of extracellular matrix, they indirectly contribute to fibrosis (Paredes et al., 2012).

It is currently acknowledged that non-alcoholic fatty liver disease (NAFLD) is the most prevalent chronic liver disease globally. Up to a third of adults in high-income nations suffer from the disorder in general, and up to 70%–80% of those who have type 2 diabetes mellitus (T2DM) also have it (Lonardo et al., 2015). In addition, even in individuals with relatively “normal” serum liver enzyme levels, patients with T2DM have an increased risk of developing more severe histological manifestations of NAFLD, such as advanced fibrosis, cirrhosis, and non-alcoholic steatohepatitis (NASH) (Gan et al., 2025; Acharya et al., 2021; Sasse et al., 1992). As a matter of fact, a recent meta-analysis has verified that over 38% of T2DM patients worldwide had biopsy-confirmed NASH, and roughly 17% of those with both NAFLD and T2DM had advanced fibrosis (Younossi et al., 2019). One herbicide that is harmful to both people and animals is called an organophosphate (OP). Numerous systems are affected by OP poisoning, including the immune system, liver, kidney, and nervous system (Abbasi et al., 2024). Research revealed that the toxicity of OPs is caused by oxidative stress, or the overproduction of free radicals, in addition to cholinesterase inhibition (Mbah Ntepe et al., 2020). Abnormal organ functioning could result from this oxidative stress. Affected levels of blood bilirubin and aminotransferases indicate impairment in metabolic, chemical, and architectural processes in the liver (Karami-Mohajeri et al., 2017). Research has verified that the primary organ impacted by OP poisoning is the liver tissue. Chlorpyrifos is a common example of an OP, which is harmful to the structure and function of the liver (Mikhail et al., 1979). When the ECM becomes less remodelled and accumulates over time, it can cause liver fibrosis by upsetting the liver’s natural architecture (Iredale, 2007). The excessive formation of ECM that results from an increased wound healing response to chronic liver injury is caused by several powerful angiogenic mediators (Rosmorduc and Housset, 2010), (Johnson and DiPietro, 2013) By releasing cytokines, the ECM can also have an indirect impact on cell activity. These include basic fibroblast growth factor (bFGF), vascular endothelial growth factor (VEGF), tumour necrosis factor-α (TNF-α), transforming growth factor β (TGF-β), platelet derived growth factor (PDGF), hepatocyte growth factor (HGF), connective tissue growth factor (CTGF) (Wight and Potter-Perigo, 2011). The primary ECM-producing cells in the wounded liver are HSCs (Kisseleva and Brenner, 2011). Together with additional cells referred to as nonparenchymal cells, parenchymal cells, or hepatocytes, compose the liver. HSCs, Kupffer cells (KCs), and liver sinusoidal endothelial cells (LSECs) are the three nonparenchymal cell types that line the walls of the hepatic sinusoids. Liver fibrosis and cirrhosis are a result of the combination of parenchymal and nonparenchymal cells in the liver. Hepatic immune responses are initiated and regulated by Kupffer cells’ phagocytic clearance of pathogens and debris as well as the release of inflammatory cytokines upon activation (Zhou et al., 2014). Immune events that activate glomerulonephritis are first, there is significant evidence that genetic and immunogenetic variables are important in determining not only those who develop a disease in response to a given stimulus but also how severe the disease is contracted, which affects the prognosis and therapy response (Rees, 1984; Rees et al., 1984). The second is that there’s growing evidence that a greater number of glomerular disorders mediated by antibodies are actually autoimmune in character than was previously thought. Both glomerular basement membrane (GBM) nephritis and systemic lupus nephritis are known to be autoimmune diseases. Membranous nephropathy (MN) (Couser and Abrass, 1988; Makker et al., 1996) IgA nephropathy (Ballardie et al., 1988; Moore et al., 1990), idiopathic crescentic glomerulonephritis (Jennette et al., 1989; Jayne et al., 1990), hemolytic uremic syndrome (Leung et al., 1988), and other types of vasculitis (Leung et al., 1988) are among the illnesses for which there is current evidence linking immune regulatory abnormalities with autoimmune processes.

### 1.2 The pathogenesis of renal fibrosis

**FIGURE 2:**
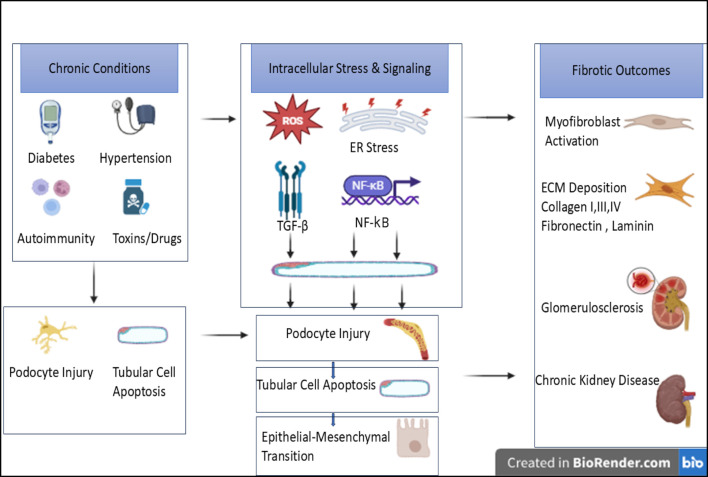
Schematic representation of shared intracellular mechanisms driving renal fibrosis in chronic kidney diseases.

Numerous factors are at play in the complicated pathophysiology of diabetic nephropathy (DN), including inflammatory, hemodynamic, and metabolic ones. Throughout the course of diabetic kidney disease, inflammatory reactions, hyperglycemia-induced oxidative damage to mitochondria, and the buildup of advanced glycation end products (AGEs) are all associated with renal injury and fibrosis (Yu et al., 2024). Diabetes complications include diabetic cardiomyopathy, diabetic nephropathy, diabetic neuropathy, and diabetic retinopathy can be brought on by long-term hyperglycemia, which can also induce pathological alterations in a number of organs and tissues. As of 2021, the International Diabetes Federation reported that 537 million adults (20–79 years old) globally have diabetes, representing approximately one in ten adults. By 2030, that figure is projected to increase to 643 million. Diabetic nephropathy affects between 30% and 40% of diabetics, according to clinical evidence. A rough estimate of the morbidity of diastolic dysfunction in diabetics is 52%–60%. Patients with diabetic nephropathy and diabetic cardiomyopathy face significant financial hardships in addition to physical complications (Zheng et al., 2024). With a rising incidence, chronic kidney disease (CKD) affects over 800 million individuals worldwide and has become a major issue. CKD is characterized by a progressive decrease of renal function over time and is brought on by blockage, diabetes, hypertension, and glomerulonephritis (Deng et al., 2024). Fibrosis is caused by the abnormal deposition of ECM produced by myofibroblasts from several sources. One of the key driving factors of fibrosis is TGF-β, which acts through both Smad-dependent and Smad-independent mechanisms (Kuppe et al., 2021; Meng et al., 2016; Ruiz-Ortega et al., 2020). As illustrated in Figure 2 Renal fibrosis can result from a number of other etiologies in addition to diabetes-related mechanisms, such as chronic hypertension, autoimmune glomerular diseases (such as lupus nephritis and IgA nephropathy), recurrent infections, obstructive nephropathy, kidney injury caused by drugs or toxins, ischemic damage, and genetic disorders. These conditions all contribute to the kidney’s progressive structural and functional decline.

## 2 Models for liver fibrosis

### 2.1 Carbon tetrachloride model

**FIGURE 3:**
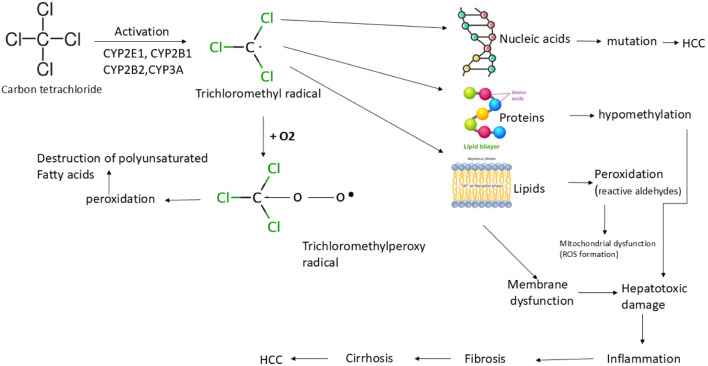
Patho biochemical sequence of events during CCl_4_-induced liver damage.

Mice can be used to model experimental liver fibrosis through hepatotoxic administration, genetic modification of gene involving fibrosis (such as Mdr2 knockout mice) or surgical procedures (such as bile duct ligation). 1. In particular, one of the most often used experimental models for causing toxin-mediated liver fibrosis is the administration of carbon tetrachloride (CCl_4_), either once or repeatedly (Scholten et al., 2015). On the other hand, prolonged ingestion of CCl_4_ results in significant hepatotoxicity, which can lead to cirrhosis, fibrosis, increased bile duct formation, and the development of hepatocellular carcinoma (HCC) (Keysser, 1976). Liver damage occurs due to the monooxygenase cytochrome P450 superfamily (CYP family) in the liver which breaks down CCl_4_ into the trichloromethyl radical (ccl3*). This radical then interacts with proteins, lipids, and nucleic acids to disrupt important cellular functions, leading to changes in lipid metabolism (steatosis and fatty degeneration) and decreased protein levels as shown in Figure 3.

Further mutations and the development of HCC are triggered by the adduct formation between (ccl_3_*) and DNA. Oxidation of (ccl_3_*) produces trichloromethylperoxy radicals (CCl_3_OO*), which further starts lipid peroxidation and polyunsaturated fatty acid degradation. As a result, there is a decrease in the cell membrane permeability of the smooth endoplasmic reticulum, cell membrane, and mitochondria. This leads to the development of widespread liver damage, which is defined by cirrhosis, inflammatory response, tissue scarring, and HCC development. There is another place where a thorough description of the pathogenetic processes within the liver as a result of CCl_4_-induced injury is provided (Weber et al., 2003). It is commonly acknowledged that mice susceptibility to CCl_4_ is highly strain-dependent as shown in the (Table 1). Specifically, FVB/N laboratory mice react with limited response to CCl_4_, but BALB/c inbred strains tend to be highly vulnerable towards the development of fibrosis. Due to the easy availability of genetically engineered mice, C57BL/6 mouse strain often exhibit moderate levels of liver fibrosis; yet, this particular strain is widely employed for fibrosis research using the CCl_4_ model. Careful strain selection is necessary to achieve consistent fibrosis induction and reliable evaluation of antifibrotic therapy since the severity of CCl_4_-induced liver injury varies greatly among mouse strains and depends on the route of delivery. In order to help with model selection, Table 1 lists frequent administration methods and strain-specific susceptibility profiles.

**TABLE 1:** Comparative susceptibility of mouse strains via various routes of administration.

Strain	Application routes	Susceptibility	References
BALB/c	IP, SC, PO	Susceptible	Bhathal et al. (1983), Hillebrandt et al. (2002), Shi et al. (1997), Walkin et al. (2013)
C57BL/6	IP	Moderate	Hillebrandt et al. (2002), Walkin et al. (2013)
DBA/2	IP	Moderate	Hillebrandt et al. (2002), Walkin et al. (2013)
FVB/N	IP	Resistant	Hillebrandt et al. (2002)
A	IP	Resistant	Hillebrandt et al. (2002)
C3H/He	IP	Resistant, high mortality (>60%)	Hillebrandt et al. (2002)
AKR	IP	Resistant high mortality (>60%)	Hillebrandt et al. (2002)

IP, intraperitoneal; SC, subcutaneous; PO, per os (via gavage).

The length of treatment and the intervals between applications can essentially control the differences in CCl_4_ responses resulting from hereditary factors and the intensity (strength) of fibrogenesis. In studies it is found that giving CCl_4_ intraperitoneally (IP) two times per week for 6 weeks, or three times per week for 4 weeks, causes severe accumulation of collagen matrix within the liver and fibrosis that, according to Desmet-Scheuer scoring, resembles human stage 3 (Desmet et al., 1994). While longer treatment can partially compensate for the lower susceptibility of strains resistant to fibrosis, short-term exposure to CCl_4_ is usually sufficient to produce fibrosis in highly susceptible strains. There are several ways to give CCl_4_, such as intraperitoneal injection, oral gavage, or inhalation, all of which have an impact on the degree and pattern of liver damage. Because intraperitoneal delivery is repeatable and has reasonable survival rates, it is most commonly used (Chang et al., 2005).

**FIGURE 4:**
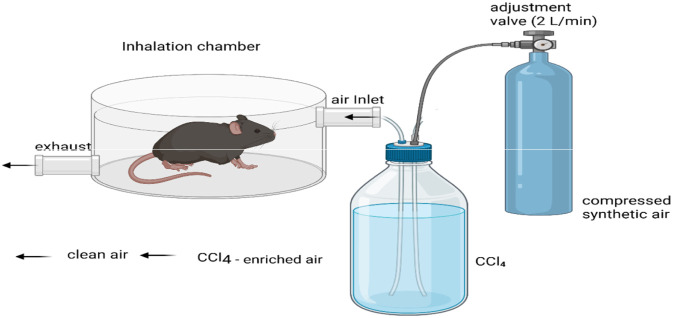
Inhalation exposure setup and procedure for CCl_4_-Induced liver damage in rodents.

Although it needs specialist laboratory infrastructure, inhalation-based exposure has been used in certain experimental scenarios, such as the production of portal hypertension, especially in rat models as shown in the (Figure 4). Despite the fact that oral gavage has been documented by a number of researchers, its use is linked to a high rate of early mortality, which limits its applicability for routine fibrosis studies (McLean et al., 1969).

#### 2.1.1 Limitations

CCl_4_ model is frequently utilized for research on liver fibrosis in rodents. However, it has some limitations, such as causing reversible fibrosis, having a narrow treatment window, and lacking a metabolic aspect. Additionally, due to its toxic properties and differences between strains, it raises safety concerns. To better reflect the intricacies of liver disease in humans, the CCl_4_ model should be paired with other fibrosis models. This approach would ensure animal welfare and improve the reliability of the results.

### 2.2 Bile duct ligation (BDL)

BDL has the potential to be carried out by double-ligating the common bile canal, either in the presence or absence of transection made at the midpoint between the ligatures (Kerfoot et al., 2006; D'Mello et al., 2009; Magen et al., 2009; Magen et al., 2010; Zarrindast et al., 2012; Nasehi et al., 2016; Tag et al., 2015a; Tag et al., 2015b). But if one knot is loose during the bile duct is dissected between the ligatures, there could be a possibility of bile leakage, which could cause fatalities and serious peritonitis. There is a low death rate when inflammatory liver damage and fibrosis are successfully generated in mice with C57BL/6 by doubly ligating the typical bile duct without transection. Fourteen days following BDL, periportal and perisinusoidal fibrosis appear, but the concentrations of liver enzymes such as ALT and AST rise to their maximum around 10–14 days (Tag et al., 2015b; Cho et al., 2020). BDL is especially useful for studying biliary fibrosis, which is characterized by periportal extracellular matrix accumulation, because it primarily causes fibrosis through persistent cholestasis and bile acid-mediated injury. However, it is not a suitable model for the slow, metabolically driven fibrogenesis seen in chronic metabolic liver diseases.

#### 2.2.1 Limitations

A method used in surgery to examine liver fibrosis, especially cholestatic liver harm, is known as the BDL model. Its limitations are that it does not adequately represent the gradual development of fibrosis seen in most human chronic liver diseases, leads to extrahepatic cholestasis, and causes the rapid progression of severe fibrosis. Due to potential issues with technical consistency and postoperative complications affecting repeatability and animal survival, the BDL model is better suited for studying biliary fibrosis rather than broader pathways of liver fibrogenesis.

### 2.3 Dimethylnitrosamine induced liver fibrosis

Dimethylnitrosamine is an effective toxin that targets the liver. According to Magee, it can damage rats’ livers and affect their metabolism and tissue distribution (Magee, 1956). Pritchard and Butler explained how it damages hepatocytes and causes cell death by apoptosis (Pritchard and Butler, 1989). Rats and dogs have been shown to develop liver fibrosis when this chemical is administered intermittently (Madden et al., 1970; Jenkins et al., 1985). Using this model, the processes and morphologic changes associated with liver fibrosis have been thoroughly studied. Early research on rats treated with DMN for 3 weeks resulted in micronodular cirrhosis without steatosis and centrilobular haemorrhagic necrosis (Jezequel et al., 1987). KCs, which are sinusoidal hepatic macrophages, were shown to be more prevalent in DMN-induced fibrotic alterations. These cells become myofibroblasts, which are the main cause of fibrosis because they produce an excessive amount of extracellular matrix (Winwood and Arthur, 1993).

Hepatotoxicity is the main way that DMN causes liver fibrosis. This causes hepatocyte apoptosis and activates sinusoidal macrophages, which then develop into myofibroblasts that produce excessive amounts of extracellular matrix. This model enables the investigation of fast cirrhosis induction within a few weeks and increasing liver damage. Hepatocyte loss and fibrous septa extension are regularly seen in histological investigation; Masson’s Trichrome staining is frequently used to visualize these findings. However, the tight dose-response relationship raises the risk of severe toxicity and mortality, and DMN shows high systemic toxicity that can impact organs including the kidneys and bone marrow. Because of these systemic effects, this model’s predictive relevance for human disease and drug testing is limited, even if it is useful for studying processes of hepatotoxicity and fast fibrosis.

#### 2.3.1 Limitations

A chemical that is utilized in research on liver damage and fibrosis is the liver fibrosis model created by DMN. This method has several disadvantages, such as producing a pattern of fibrosis that is both peri-central and widespread, along with significant systemic toxicity that affects organs including the kidneys and bone marrow. The sharp dose-response relationship and narrow dosing range of this model increase the risk of severe toxicity and mortality. Additionally, it does not display features such as bile duct growth or steatosis.

### 2.4 Hepatic stellate cells (HSC) in culture

The precise processes by which HSCs, or hepatic stellate cells, aid in liver regeneration especially inside the intact organ *in vivo*, are still unknown (Omenetti et al., 2008). The research of cellular activation pathways, such as their differentiation into myofibroblasts and excessive extracellular matrix formation during fibrosis, is made possible by the broad availability of HSC culture models, especially when employing human cells. These models are especially useful for drug screening and testing of antifibrotic drugs because they offer crucial insight into the mechanisms causing hepatic fibrogenesis. HSCs can be cultured *in vitro* using DMEM supplemented with 10% fetal bovine serum (FBS) and antibiotics in plastic dishes under carefully monitored conditions (Schnabl et al., 2002). Their morphology and activation status have been observed using α-SMA staining and light microscopy (Benten et al., 2005). Experiments including transduction, radiolabelling, and transplantation have shown how HSCs integrate and react *in vivo*, demonstrating their behavior during the advancement of fibrosis; nonetheless, the biological insights gained outweigh these methodological subtleties. HSC cultures have contributed to the understanding of how hepatic injury signals (such as oxidative stress and DAMPs) trigger HSCs, their subsequent proliferation, and ECM deposition following experimental fibrosis induction in animal models such as CCl_4_ and Bile Duct Ligation (BDL) (Lerche-Langrand and Toutain, 2000). By providing regulated settings to investigate biological pathways and possible therapeutic interventions, these *in vitro* models supplement *in vivo* research.

**FIGURE 5:**
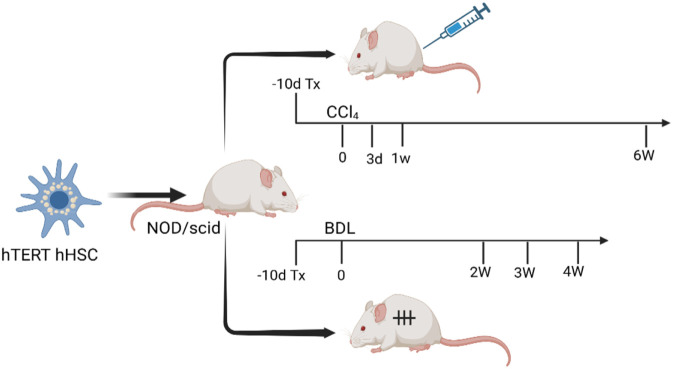
Schematic Overview of hHSC Transplantation and Liver Fibrosis Induction Using CCl_4_ and Bile Duct Ligation Models.

Schematic Overview of hHSC Transplantation and Liver Fibrosis Induction Using CCl_4_ and Bile Duct Ligation Models shown in Figure 5.

#### 2.4.1 Limitations

An effective *in vitro* system for studying liver fibrosis, particularly how it activates, is HSCs. However, this system has limitations such as changes in cell characteristics, unplanned activation, and absence of a detailed liver microenvironment. Different results may arise from variations among species and difficulties in maintaining primary human HSCs. Despite these limitations, HSC cultures are useful for drug-related and mechanistic studies, but findings should be validated in models that better mimic physiological conditions.

### 2.5 Liver slice system

Because they allow for investigating HSC activation and liver fibrosis *in vitro* in a multicellular system that maintains connections between cells and the extracellular matrix, along with well sliced liver segments have recently drawn attention as a potential model for these studies. Nevertheless, there are still few studies using liver slices as a method to examine fibrogenesis and HSC activation (Olinga and Groothuis, 2001; Elferink et al., 2004). The ability to investigate liver function *in vitro* in a multicellular setting is made possible by the existence and functionality of every type of liver cell. Hepatocytes KCs (Neyrinck et al., 2004; Oudar et al., 1998), endothelial cells (Beljaars et al., 2001), and HSC throughout incubation, according to numerous investigations into the health and performance of the different cell types found in liver slices (Van de Bovenkamp et al., 2005; Van de Bovenkamp et al., 2006; Berthiaume et al., 1996). Crucially maintains the cells’ original extracellular matrix, their relative quantity, and their orientation with respect to other cells contrast to coculture models of isolated cells. Furthermore, when blood flow is reduced, the liver’s acinar structure is preserved, however it is unclear for how long zonal distinctions between cells are preserved. The ECM present in liver slices is made up of various collagens fibers, glycoproteins, and proteoglycans molecules. It encircles the many types of liver cells and hence maintains the liver’s coherence while also controlling cellular activity. The ECM’s makeup can affect the liver cells’ ability to differentiate, proliferate, and activate (Imai et al., 2000; Shakado et al., 1995; Friedman, 2003). As was previously indicated, the widespread consensus is that the primary factor in the development of hepatic fibrosis is HSC activation. As a result, research on the suitability of liver slices for fibrosis research was mostly concentrated on HSC. HSC become activated and differentiate into cells resembling myofibroblasts during liver fibrosis see as described in Table 2. These cells are the primary creators of the extracellular matrix excess that causes liver failure (Diehl and Day, 2017).

**TABLE 2:** Comparison of experimental models for studying liver fibrosis: key pathological features, biochemical changes, and disease stages.

Model	Type	Key pathological features	Main biochemical and morphological alterations	Disease stage represent
Carbon tetrachloride (CCL_4)_	Chemical (*in vivo*)	Hepatocyte necrosis, steatosis, bridging fibrosis	↑ALT/AST, oxidative stress, collagen I/III deposition	Fibrosis→Cirrhosis→HCC
Bile duct ligation (BDL)	Surgical (*in vivo*)	Bile duct proliferation, periportal fibrosis	↑ bile acids, cholestasis, inflammatory infiltration	Biliary fibrosis
Dimethylnitrosamine (DMN)	Chemical (*in vivo*)	Centrilobilar necrosis, fibrous septa	↑AST, hepatocyte loss, micronodular architecture	Fibrosis →Cirrhosis
Hepatic stellate cells (HSC_S)_	*In vitro*	Myofibroblast activation, ECM accumulation	↑ α-SMA, collagen synthesis	Cellular mechanisms of fibrosis
Liver slice system	*Ex vivo*	Preserved tissue architecture	Early ECM remodelling, HSC activation	Early fibrogenic responses

#### 2.5.1 Limitations

A system that keeps liver fibrosis and medication reactions outside of a living body is the liver slice culture method. It has several limitations such as inadequate blood flow, challenges in attracting immune cells, overall body effects, restricted fibrotic reaction, poor oxygen and nutrient spread, and short survival periods. Differences in the thickness, quality, and preparation techniques of the slices can affect the reliability and consistency of data. Liver slice systems are constrained by short survival times and low immune cell recruitment, although offering important *ex vivo* insights into hepatic physiology and fibrosis. By combining liver slices with supportive technologies such perfusion bioreactors, which sustain the flow of nutrients and oxygen, or co-culture systems that include immune cells to more closely resemble *in vivo* interactions, these restrictions can be overcome. Furthermore, the physiological relevance and translational potential of *ex vivo* liver models can be improved by merging liver slices with microfluidic systems (organ-on-a-chip) to improve survivability, enable dynamic monitoring, and permit immune cell infiltration.

For a complete understanding of how liver fibrosis develops, liver slice cultures are most effective when used alongside *in vivo* models.

## 3 In silico models in liver fibrosis

Recently, *in silico* (computational) models have become useful tools in liver fibrosis research for ranking possible antifibrotic medication candidates in addition to *in vitro*, *ex vivo*, and *in vivo* methods. In order to combine multi-omics data and simulate important fibrogenic processes, such as TGF-β/Smad signaling, inflammatory cascades, and hepatic stellate cell activation, systems biology-based models and network pharmacology techniques are employed. Predicting drug-target interactions, hepatotoxicity, and therapeutic efficacy is further aided by machine-learning algorithms and molecular docking studies, which lowers the expense of experiments and the need for animals. These computational techniques support experimental models of liver fibrosis and make it easier to choose potential drugs for further validation.

In liver fibrosis research, particular *in silico* implementations have shown definite predictive and translational usefulness beyond these broad frameworks. Specifically, by combining cellular interactions, cytokine signaling, and tissue mechanics, agent-based modeling approaches have been employed to simulate the multiscale evolution of liver fibrosis. Using the SPARK framework, a liver fibrosis agent-based model (LFABM) effectively replicated important histology characteristics of increasing tissue stiffness and fibrosis progression. Crucially, experimental data from rats with CCl_4_-induced liver fibrosis were used to validate model predictions. The model’s usefulness for hypothesis testing and preclinical evaluation of antifibrotic medicines was further demonstrated by using it to investigate possible antifibrotic techniques *in silico*, such as TNF-α inhibition and manipulation of Kupffer cell morphologies see described in Table 3 (Dutta-Moscato et al., 2014).

**TABLE 3:** Comparative overview of experimental models used in the preclinical evaluation of liver antifibrotic therapies.

Model	Advantages	Disadvantages	Suitability for drug evaluation
CCl_4_	Well-characterized, reproducible, mimics toxin-induced fibrosis, allows dose/time control	Reversible fibrosis, toxic, strain-dependent, narrow treatment window	Suitable for testing hepatoprotective and antifibrotic drugs targeting oxidative stress and inflammation
Bile Duct Ligation (BDL)	Rapid induction of biliary fibrosis, mimics cholestatic injury	Technically challenging, rapid progression unlike chronic human disease, extrahepatic cholestasis	Best for biliary-targeted therapies, limited for general antifibrotic drug testing
Dimethylnitrosamine (DMN)	Produces cirrhosis, mechanistic studies possible	High systemic toxicity, mortality risk, lacks bile duct growth	Useful for mechanistic drug studies but limited for chronic therapy evaluation
Hepatic Stellate Cell (HSC) culture	Human cells, controllable environment, mechanistic studies	Lacks full liver microenvironment, changes in cell phenotype	Ideal for *in vitro* drug screening and pathway studies before *in vivo* testing
Liver slices (precision-cut)	Maintains multicellular interactions and ECM, preserves cell orientation	Short lifespan, poor oxygen/nutrient diffusion, no systemic effects	Useful for short-term drug testing, mechanistic studies, preclinical screening
In silico models	High-throughput, low cost, integrates multi-omics	Simplified, predictive only, cannot replace *in vivo*	Excellent for initial candidate drug screening and predicting hepatotoxicity

A variety of experimental approaches are used in the liver fibrosis models under discussion, each with special benefits and disadvantages. Toxin-induced models, such as CCl4, are useful for testing antifibrotic drugs because they reliably cause fibrosis and mimic key clinical features. While chemical models like DMN and cell-based systems offer cellular mechanistic insights, bile duct ligation (BDL) models are useful for studying cholestatic fibrosis. Additionally, *in silico* models are used to predict the toxicity and efficacy of drugs. The choice of models should be in line with the goals of the research: *in vitro* and computational methods are superior for mechanistic research or high-throughput screening, whilst *in vivo* models are better suited for translational studies. The complexity of human liver fibrosis is more fully represented and experimental reliability is increased when many models are used.

## 4 Models for renal fibrosis

### 4.1 Unilateral ureteral obstruction model

**FIGURE 6:**
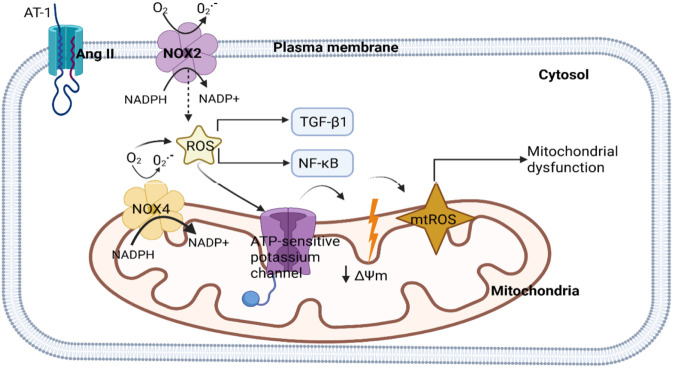
Illustration of NOX and mitochondria-dependent ROS signaling triggered by angiotensin II.

An *in vivo* model called unilateral ureteral obstruction (UUO) can be created quickly and simulates the renal fibrosis linked to chronic obstructive nephropathy (Martínez-Klimova et al., 2019). The UUO is made up of a silk thread tied around the left ureter. The non-ligated (contralateral) kidney is referred to as the non-obstructed kidney in this paradigm, whereas the ligated ureter kidney is called the obstructed kidney (Martínez-Klimova et al., 2019; Ucero et al., 2014). Following UUO, angiotensin II (Ang II) is produced as a result of urine stagnation, which raises hydrostatic pressure and activates the renin-angiotensin system (RAS). ROS are created when nicotinamide adenine dinucleotide phosphate (NADPH) oxidases (NOXs) are activated by Ang II. Production of ROS causes oxidative stress, inflammation, and eventually necrosis or apoptosis, which kills cells as in Figure 6 (Xia et al., 2018). Additionally, these pathways cause resident fibroblasts in the kidney to become myofibroblasts, which then activate ECM to replace lost epithelial cells and accelerate the progression of fibrosis (Xia et al., 2018; Checa and Aran, 2020). Molecular oxygen and nitrogen make up molecules known as ROS (Sies and Jones, 2020). They comprise both non-radical and free radical species. Non-radical species include hydrogen peroxide (H2O2), peroxynitrite (ONOO−), and organic hydroperoxides (ROOH). Examples of free radical species include hydroxyl radical (OH), superoxide anion radical (O2), nitrogen dioxide (NO2), and nitric oxide (NO) (Sies, 2017). H2O2 and NO are the main redox signalling agents in cells, and It is known that ROS mediate cellular signaling (Sies, 2017; Holmström and Finkel, 2014; Cruz‐Gregorio and Aranda‐Rivera, 2021; Wani et al., 2014) Because ROS induce PTMs found in proteins containing residues of redox-sensitive amino acids, they affect several signalling pathways, controlling the structure, function, and location of proteins (D'Autréaux and Toledano, 2007; Shen et al., 2016). In kidneys subjected to unilateral ureteral obstruction mechanical stretching is linked to RAS activation, which can lead to the advancement of various mechanisms like vasoconstriction, which might result in renal injury. Ang II triggers redox-sensitive signalling through the transcription factor NF-κB and the cytokine TGF-β1. Accordingly, TGF-β1, Smad2, and Smad3 are upregulated when Ang II is applied to normal rat kidney fibroblast (NRK49F) cells, which results in the overexpression of fibrosis indicators such α-Smad, fibronectin, and collagen I (Iaccarino et al., 2005; Wang et al., 2005). Renal fibrosis is thought to be triggered by a number of immune mediators including cytokines, chemokines, and growth-promoting factors, although TGF-β is thought to be the most powerful and common profibrogenic cytokine. This is because it has the ability to prevent ECM degradation while simultaneously inducing fibroblast and myofibroblast synthesis of ECM (Iglesias-De La Cruz et al., 2001). TGF-β1 is activated by ROS. *In vitro*, mesangial cells stimulated with H_2_O_2_ increased TGF-β1, resulting in an increase in collagen isoforms I, III, and IV, as well as fibronectin, a key structural matrix component (Xu et al., 2014). The UUO kidneys show signs of TGF-β1 pathway activation after a period of 2 days of blockage. Levels of TGF-β1 mRNA and its corresponding protein noticeably rise on the seventh day and peak on day fourteen after obstruction (Li et al., 2010; Zhang et al., 2018; Aranda-Rivera et al., 2021).

After binding onto the angiotensin type 1 (AT1) receptor, Ang II stimulates NOXs (NOX2 and NOX4), which increase the formation of ROS. The NOXs’ generation of ROS stimulates the expression of nuclear factor kappa-light-chain enhancer of activated B cells (NFκB) and TGF-β1. Additionally, ROS promotes the activation of the ATP-sensitive potassium channel in the mitochondria, which favours mitochondria depolarization (↓ΔΨm), resulting in the formation of mitochondrial ROS (mtROS) and mitochondrial malfunction (Aparicio-Trejo et al., 2019). TGF-β1 is activated by ROS that mitochondria generate. Antioxidants that target the mitochondria, such curcumin, mitoTEMPO, mitoQ, and N-acetylcysteine (NAC), reduce the nuclear translocation and upregulation of Smad2/3 and TGF-β1 *in vitro*. The latter may result from the suppression of the transcription of the TGF-β1 gene, which includes α-SMA and connective tissue growth factor (CTGF), indicating that mtROS is necessary for their transcription (Jain et al., 2013; Liu et al., 2017). Additionally, in UUO kidneys, the elevation of the protein located in the mitochondrial membrane, ROS is produced by reactive oxygen species modulator 1 (ROMO1), which triggers TGF-β1/Smad2/3 signaling, leading to a rise in, fibronectin, collagen, and vimentin and a decrease in E-cadherin (Liu et al., 2011). The effects of ROS were lessened by NAC treatment and ROMO1 protein knockdown (Liu et al., 2011). These findings imply that in the UUO model, mtROS generation stimulates TGF-β1 activation to cause fibrosis.

#### 4.1.1 Limitations

The UUO model is often utilized to study kidney fibrosis due to its rapid fibrotic changes and reproducibility. However, it has significant disadvantages, including a complete and instant blockage of urine flow, which does not accurately reflect the progression of actual CKD. Additionally, this model is not functional since it leads to fibrosis as a result of inflammation and mechanical stress—conditions that do not occur in clinical CKD cases. Moreover, its inability to replicate systemic hypertension and proteinuria restricts the therapeutic relevance of the model.

### 4.2 Subtotal nephrectomy

Renal fibrosis is significantly impacted by subtotal nephrectomy (STNx). According to studies, STNx hastens the development of renal fibrosis following myocardial infarction (MI) (Liu et al., 2013; Layton et al., 2017). STNx models are useful for researching CKD because they can cause renal fibrosis in mice without causing cardiac pathology (O’sullivan et al., 2019). Additionally, studies show that cardiac-specific lack of the cardiokine follistatin-like 1 intensifies renal damage following STNx, emphasizing the interaction of the kidney and heart in renal fibrosis (Yang et al., 2010). These findings emphasize the importance of STNx in comprehending the pathophysiological modifications in renal fibrosis and its reciprocal relationships with cardiac dysfunction in conditions including cardiorenal syndromes and end-stage renal disease. Rat and mouse 5/6 nephrectomy (PNx) is suitable model associated with renal failure following renal function loss observed in humans (Lim et al., 2014; Fernandes-Charpiot et al., 2016). Traditional PNx involves excising two-thirds of the remaining kidney after removing one kidney, which can result in significant renal haemorrhage and infection and raise the risk of kidney failure 1 week later. Recent reports describe a novel technique for PNx that mimics a traditional PNx by ligating renal artery branches (Gagnon and Gallimore, 1988). Nevertheless, this approach necessitates micromanipulation and is impractical for mice. Electrocoagulation is another often used this technique that stops bleeding after surgery (Zou et al., 2013; Liu et al., 2014; Tan et al., 2019). Using electrocoagulation to halt the bleeding, the kidney on one side is removed first, followed through the extraction of the kidney’s superior and inferior poles. Even though this technique can lessen renal bleeding, it still results in approximately 30 and 40 percent of the mice dying, and it necessitates a specific electrocoagulation device, which raises the expense and sophistication of the research. In order to minimize renal bleeding and infection during surgery, as well as animal mortality and operation complexity, we aim to optimize the current PNx. Interestingly, our pre-experiments showed that direct ligation on the kidney is able to produce necrotic damage affecting both the superior and inferior poles of the kidney, providing a way to improve the standard PNx. In order to replicate the procedures using this straightforward and user-friendly surgical technique, as well as to confirm its impact regarding kidney function and the development concerning kidney fibrosis, this study was conducted. Additionally, its potential to replace the traditional PNx in the study of CKD was investigated (Wang et al., 2000).

#### 4.2.1 Limitations

A commonly used *in vivo* method for studying progressive kidney scarring and CKD is the STNx in animal subjects. This technique simulates many features of human chronic kidney disease, such as the loss of nephrons, glomerulosclerosis, elevated protein levels in urine, and high blood pressure. However, there are some downsides, like the complexity of the surgery, unpredictability in results, and the risk of causing stress and inflammation in subjects. Additionally, since this model is irreversible, it cannot be used to explore treatment options aimed at stopping or reversing fibrosis. Moreover, the time required for fibrosis to develop may be a limitation for researchers seeking rapid outcomes. For a better representation of the various forms of kidney disease in humans, the STNx model is most effective when used alongside other models.

### 4.3 Adriamycin-induced nephropathy

Nephropathy brought on by Adriamycin (ADR) is the most commonly used prototype for researching human primary focal segmental glomerulosclerosis (FSGS), a common mechanism for damage to podocytes and glomerular impairment of function that causes renal failure and damage (Pippin et al., 2009; Bryant et al., 2022). The historical classification of C57BL/6 mice, the most prevalent genetically engineered strain, as ADR-resistant limits the applicability related to this reverse genetics’ model (Vallon et al., 2006). It is commonly recognized that adenosine protects cells in stressful conditions like ischemia, hypoxia, and inflammation (Kim and Dryer, 2022). In the normal kidney, adenosine concentration rises significantly in response to renal ischemia, hypoxia, and inflammation (Egger et al., 2015). The development of glomerulosclerosis, tubulointerstitial inflammation, and fibrosis are characteristics of the majority of kinds of CKD (Manabe et al., 2001). ADR is an anti-neoplastic drug that induces oxidative stress and affects the water and urea transporters in the renal medulla, among other processes that lead to nephrotoxicity (Hahn et al., 2004). Eight mice each age group eight in the illness and control groups of female Balb/C mice aged five and 12 weeks were employed. The mice were given an ordinary lab diet and unlimited access to water. ADR (20 mg/kg) was injected intravenously once into the tail vein of each mouse after their urine had been collected for 24 h utilizing metabolic cages. The control mice received the same amount of saline. On day 12, the mice were given ether anaesthesia following a second urine collection, and both kidneys were quickly removed utilizing cardiac catheterization and ice-cold phosphate-buffered saline (PBS) perfusion. Using the Bradford method, the amount of protein in urine samples taken both before and after AD injection was determined (Li et al., 2006). The damage to the kidneys begins after one to 2 weeks and continues steadily for a few weeks. Renal functions decline and ECM proteins progressively build up in glomeruli and tubulointerstitial spaces throughout this time. After the duration of the study, the animals are killed, and the kidneys are examined utilizing the previously mentioned immunohistochemistry and gene expression studies for tubular damage, inflammation, and fibrosis. A number of medications, including the angiotensin converting enzyme inhibitor captopril, the angiotensin receptor antagonist losartan, and several kinase inhibitors, have been assessed using this model (Kim et al., 2004; Mansour et al., 1999; Min et al., 2016). We used the Trizol reagent (Poration, Indianapolis, IN, USA) and SYBR Green technology to extract total RNA from experimental cells. For a total reaction volume of 20 μL, 10 μL SYBR Green master mix, 1 μL of RNA (equivalent with 50 ng of total RNA), and 900 nM concentrations of both forward and reverse primers were added to 96-well PCR plates for real-time polymerase chain reaction. Reverse transcriptase PCR was carried out in real time for 5 minutes at 95 °C and 10 minutes at 50 °C (Kim and Dryer, 2022).

#### 4.3.1 Limitations

A model that is utilized to study glomerulosclerosis and kidney fibrosis, particularly concerning kidney injury caused by chemotherapy, is nephropathy induced by ADR. Despite its applications, this model has several limitations, such as toxicity to cells, oxidative stress, and damage to DNA, which differ from the ways kidney diseases affect humans. In addition, it leads to rapid onset of acute kidney damage and fibrosis, which does not reflect the typical progression of kidney disease in people. Moreover, this model fails to accurately represent tubulointerstitial fibrosis, focusing instead on damage to the glomeruli. It can lead to further complications and is harmful to the body.

### 4.4 Protein overload rat model

Renal fibrosis has been studied using a protein overload rat model in several publications. In the paradigm, rats are given albumin overload (AO) to induce proteinuria, which results in tubulointerstitial disease, which is characterized by inflammation and fibrosis (Fang and Yang, 2018). Furthermore, albumin overload-induced nephropathy has been linked to the (pro)renin receptor (PRR), and PRR antagonists have been shown to have Reno protective effects by blocking the intrarenal renin-angiotensin system (Secher et al., 2018). Additionally, it has been demonstrated that renal hypertrophy and fibrosis develop in a surgically generated rat model of non-insulin dependent type 1-like diabetes, involving pancreatectomy and uninephrectomy. This offers a fresh approach for researching therapeutic candidates for diabetic nephropathy (Klein et al., 2011). It has also been shown that incremental load training in old mice improves renal fibrosis through activating autophagy and modulating the TGF-β1/TAK1/MKK3/p38MAPK signaling pathway (Dryer et al., 2019). Cationic channels are transient receptor potential-6 (TRPC6) channels with permeability to calcium ions and are expressed in a variety of cell types such as mesangial cells as well as podocytes found in renal glomeruli (Ilatovskaya et al., 2014). Ca^2+^ signaling is mediated by these podocyte channels as a reaction to signals provided by endocrine and circulatory factors, including angiotensin II and ATP (Anderson et al., 2014; Roshanravan and Dryer, 2014; Anderson et al., 2013), moreover, they have the ability to activate in response to mechanical stimuli *in vivo* (Gyarmati et al., 2022) as well as *in vitro*, such as rises in intraglomerular capillary pressure (Winn et al., 2005). Patients suffering from familial types of focal and segmental glomerulosclerosis (FSGS) have mutations in the gene that codes for TRPC6 channels (Reiser et al., 2005; Kim et al., 2018). The detailed description of the Sprague-Dawley strain Trpc6wt/wt and Trpc6del/del rats utilized in these investigations was earlier published (Kim et al., 2019). For 28 days, the animals received intraperitoneal (i.p.) injections of 1.7 g/day of sterile normal saline dissolved with bovine serum albumin (BSA). The saline vehicle was given in equal amounts to the control animals. Making use of a commercially available ELISA test (Exocell Inc., Philadelphia, PA, United States), urine albumin was quantified in 24-h samples of urine before BSA injections, 14 days after injections of albumin, as well as at the close of 28- day duration. The day following the final BSA injection, the animals were sacrificed by inhaling CO2 and then dislocating their cervical vertebrae. The kidneys were removed, weighed, and used for histological and biochemical examinations. Blood was also collected. The next step involved utilizing an Arbor Assays kit (San Jose, CA, United States) to measure blood urea nitrogen (BUN). Animal kidney slices from each group were embedded in paraffin, fixed with a 10% buffered formalin immersion, and stained in 4 µm sections using Masson’s trichrome or Periodic Acid-Schiff’s (PAS) techniques, as previously detailed in detail (Kim et al., 2019; Kim and Dryer, 2021) and an observer who was blind to the treatment group or genotype assessed sections dyed with a semi-quantitative scale using Masson’s approach (Star, 1998). In short, tube atrophy, tubular dilatation, interstitial fibrosis, protein casts, and interstitial infiltrates were detected in at least 20 tubulointerstitial areas per animal under ×20 objectives. Each section was assigned a rating of (0) normal, (1) mild change, (2) moderate change, or (3) severe change based on these criteria.

#### 4.4.1 Limitations

A helpful tool for researching kidney illness, especially proteinuria-induced kidney damage and fibrosis, is the protein overload rat model. It involves giving the kidneys large amounts of proteins, including albumin, which damages the kidneys. The intricacy of kidney illnesses in humans, the quick development of fibrosis, and the absence of systemic comorbidities are some of the model’s drawbacks. Furthermore, the direct delivery of high-protein loading in the model could not be an accurate representation of the processes involved in actual illness. Furthermore, because rats and humans may react differently to protein-induced injury, species differences may restrict the model’s translational utility. In order to give a more thorough understanding of renal fibrosis, the model is most useful when combined with other models.

### 4.5 Renal ischemia/reperfusion-induced fibrosis

AKI is mostly caused by ischemia/reperfusion injury, which is also linked to delayed graft function and a higher risk of acute rejection following kidney transplantation. However, recent research has indicated that the development of chronic kidney disease may be significantly influenced by post-inflammatory renal scarring brought on by ischemia/reperfusion injury (Forbes et al., 2000; Yun et al., 2009). Aortic bypass surgery, kidney graft procedure, partial kidney removal, angioplasty of the renal artery, sepsis, fluid accumulation in the kidney, non-emergency urologic surgery procedures, cardiopulmonary bypass, surgical bypass of the aorta, liver grafting, the use of blood vessel constrictors medications, and specific states of reduced blood pressure are among the clinical scenarios in which ischemia is the primary cause of AKI (Barri et al., 2009; Ko et al., 2008). Prior to manipulation, 150–200 g male Sprague Dawley rats were allowed unrestricted access to food and water. Rats were housed at 25 °C with a 12-h light/dark cycle, and they were given unlimited access to water and normal rat food (Weng et al., 2012). Six rats per group, or six total, were randomly assigned to one of three groups: (1) ischemia-reperfusion injury group, where the kidneys underwent 45 min of ischemia and subsequent reperfusion; (2) ischemic postconditioning (IPO) group, where six cycles of 10-s reperfusion alternating with 10-s ischemia were applied after 45 min of ischemia; and (3) sham group. After 12 weeks of reperfusion, the rats were sacrificed. A fully maintained anaesthetic was used to remove the left kidney and draw blood through an inferior vena cava puncture. The kidneys were removed, frozen right away, and preserved at 80 °C for later uses. They were also fixed in 10% phosphate-buffered formalin (Mauro et al., 2005). Kidneys were paraffin embedded, sectioned and preserved in 10% neutral-buffered formalin 5-mm-thick sections in accordance with normal protocol for histologic preparations. The sections underwent a progressive process of deparaffinization and hydration, followed by staining with haematoxylin and eosin (H&E) and Masson’s trichrome. An experienced renal pathologist carried out the morphologic evaluations in a blind manner. Using the previously reported method of estimating the percentage of the afflicted area fraction-ten fields per section at 200 magnification-long-term tubulointerstitial injury was assessed (Bikbov et al., 2020).

### 4.6 Immune-mediated glomerulosclerosis: the role of anti-thy 1 antibody

One of the key pathomechanisms in the onset and CKD progression is a worldwide community health issue that impacts at least 9.1% of the global population, is kidney fibrosis (Chronic Kidney Disease Prognosis Consortium et al., 2010). CKD is associated with an early vascular aging phenotype which significantly increases the risk of cardiovascular morbidity and death in those who have it (Go et al., 2004; Ebert et al., 2020; Lousa et al., 2020; Ebert et al., 2022). Whatever the cause of CKD, oxidative stress, hypoxia, and persistent inflammation are the main causes of renal fibrosis, which affects every kidney compartment and eventually results in glomerulosclerosis and arteriosclerosis as well as the permanent loss of kidney function (Ebert et al., 2022; Ebert et al., 2021; Saalbach and Anderegg, 2019). A glycosylphosphatidylinositol anchor attaches the tiny, highly glycosylated protein known as Thymocyte Differentiation Antigen-1 (Thy-1, CD90) to the plasma membrane’s outer leaflet (Wang et al., 2006). Fibroblasts, neurons, glomeruli cells, active mouse T cells, mesenchymal stem cells, hematopoietic stem cells, and microvascular endothelial cells are among the tissues on which it is expressed. Thy-1 membrane receptors have been identified, including β2, β3, β5 integrins, CD97, and syndecan-4. Furthermore, Thy-1 interacts with molecules inside the same cell’s membrane (Wang et al., 2006).

Male Wistar rats weighing 150–180 g was housed in a room maintained at a constant temperature with a 12-h light/dark cycle. To allow for acclimatization, the rats were fed a regular protein diet for at least 3 days prior to the experiment’s commencement. Every two to 3 days, the animals’ body weight and food and water intake were recorded, and they were visited daily. Three days following a unilateral nephrectomy, as previously described, an intravenous injection of mAb 1-22-3 monoclonal antibody, administered at 5 mg/kg body weight in PB, pH 7.4 was used to produce anti-thy1-induced chronic-progressive glomerulosclerosis (cGS) (Peters et al., 2004). Within the following 24 h, the mAb 1-22-3 antibody recognizes and binds to thy1-like antigen present on mesangial cells, resulting in a rapid lysis of mesangial cells involving complement activation and NO (Wang-Rosenke et al., 2013).

Since the glomerular conditions improved in around 4 weeks in mice with two kidneys, the uninephrectomy conducted prior to anti-thy1 antibody administration contributes to the long-term progression of cGS. The only injection used on animals used as controls, including those either with or without uninephrectomy, was identical quantities of PBS. As controls, there were four non-nephrectomised animals receiving PBS injections (2-K Control) and four nephrectomised animals injected with PBS (1-K Control). The diseased animals were stratified and assigned to the uninephrectomy, anti-thy1-injected animals, no treatment (cGS, n = 11) and uni-nephrectomised, anti-thy1-injected animals treated with Imatinib (cGS + Imatinib, n = 11) groups based on the precise 24-h proteinuria observed 1 week following anti-thy 1 antibody administration. In order to prevent anti-thy1 antibody from interfering with the disease-inducing process therapy began 7 days after the antibody infusion.

#### 4.6.1 Limitations

Despite its limitations, the anti-Thy-1 antibody model plays a valuable role in the study of immune-mediated glomerulosclerosis and kidney fibrosis. This model inflicts damage by initiating an immune response specific to the Thy-1 antigen, which does not extend to various kidney diseases seen in people. Rather than replicating the systemic effects present in human kidney conditions, it mainly focuses on injuries to the glomeruli. Furthermore, it does not adequately explain tubulointerstitial fibrosis. Variations among strains and differences between species limit its applicability. It is recommended to use multiple models to gain a more comprehensive understanding of kidney fibrosis.

### 4.7 *In silico* models in renal fibrosis

Similarly, *in silico* modeling methods are being used more often in renal fibrosis research to augment traditional experimental models. One of the main pathophysiological causes of chronic kidney disease is renal fibrosis, which is primarily caused by hyperactivation of the TGF-β/TGFβR-1/Smad signaling pathway. A thorough *in silico* drug-discovery methodology was used to screen natural flavonoids that target TGFβR-1 in order to find safer antifibrotic compounds. After screening 51 flavonoids using ADME/T prediction (QikProp), molecular docking (Glide/Maestro), binding free-energy calculations (MM-GBSA), and 200-ns molecular dynamics simulations (Desmond, Schrödinger suite), 31 drug-like candidates were found. Compared to the reference medication linagliptin, epicatechin, fisetin, and luteolin were found to be the top leads with better binding affinities and more stable protein–ligand interactions using docking and stability analyses. These substances effectively inhibited TGFβR-1 by continuously engaging important active-site residues and maintaining stable conformations during simulations. This case study shows how *in silico* modeling can effectively rank antifibrotic medication candidates, lessen the burden of experimentation, and direct further *in vitro* and *in vivo* validation for treatments for kidney fibrosis (Rahman et al., 2022).

Computational frameworks that incorporate transcriptome and proteome information can replicate important profibrotic events in kidney cells, including TGF-β signaling, extracellular matrix remodeling, epithelial–mesenchymal transition, and inflammatory responses. Network-based research and machine learning methods are used to identify key regulatory nodes in chronic kidney disease and predict antifibrotic drug responses. The translational value of research on renal fibrosis is increased by these techniques, which aid in the development of hypotheses, medication prioritization, and experiment burden reduction. Several experimental approaches for examining kidney damage and fibrosis are included in the renal fibrosis models that are being discussed. For evaluating increasing fibrosis and possible treatments, common models like UUO and STNx are crucial. While ischemia/reperfusion and immune-mediated models deal with acute circumstances, other models, such as adriamycin-induced nephropathy and protein overload, concentrate on particular injury processes. Kidney slices and grown cells are examples of *in vitro* and *ex vivo* systems that provide mechanistic insights and alternatives for first drug testing. Predicting drug interactions and toxicity is another benefit of computational methods. The model selection should be in line with the goals of the study; *in vitro*/*in silico* techniques are appropriate for mechanistic investigations, while *in vivo* models are preferred for translational research see Table 4. The reliability and translational relevance of results about human kidney fibrosis are improved by combining many models.

**TABLE 4:** Commonly used experimental models of chronic kidney disease and renal fibrosis: advantages, disadvantages and applications in drug evaluation.

Model	Advantages	Disadvantages	Applications
Unilateral Ureteral Obstruction (UUO)	Rapid fibrosis induction, reproducible, well-characterized	Acute obstruction, does not mimic human CKD progression, lacks proteinuria/systemic features	Suitable for mechanistic studies and testing antifibrotic agents targeting TGF-β/ROS pathways
Subtotal Nephrectomy (STNx/5/6 PNx)	Mimics progressive CKD, nephron loss, glomerulosclerosis, hypertension	Surgical complexity, variable outcomes, irreversible	Suitable for long-term studies of CKD therapies and antifibrotic drugs
Adriamycin (ADR)-induced nephropathy	Models’ glomerular injury, podocyte damage, and glomerulosclerosis	Rapid onset, acute toxicity, does not fully replicate CKD progression	Useful for testing drugs targeting glomerular injury and chemotherapeutic nephrotoxicity
Protein Overload (Albumin) Model	Induces tubulointerstitial fibrosis via proteinuria	Rapid fibrosis, lacks systemic comorbidities, species differences	Useful for testing antifibrotic therapies targeting tubulointerstitial injury and proteinuria
Ischemia/Reperfusion (I/R) Injury	Models AKI leading to fibrosis, clinically relevant to transplant or surgery	Acute injury model, may not reflect chronic CKD	Suitable for studying therapies preventing post-AKI fibrosis
Anti-Thy1 antibody-induced glomerulosclerosis	Immune-mediated fibrosis, reproducible in Wistar rats	Restricted to glomerular injury, not all CKD types	Useful for testing immunomodulatory or antifibrotic therapies targeting glomerular injury

## 5 Conclusion

Global health is significantly impacted by the progressive and complex pathological process of organ fibrosis, especially in the liver and kidneys. With a focus on the functions of hepatic stellate cells, myofibroblast activation, oxidative stress, and immune-mediated signalling, this review has brought attention to the complex cellular and molecular mechanisms underlying hepatic and renal fibrosis. Diverse *in vitro* and *in vivo* experimental models, including bile duct ligation, UUO, adriamycin nephropathy, and CCl_4_-induced liver injury, have been used to improve our understanding of the course of fibrosis and the response to treatment. In models of both liver and kidney fibrosis, immune cells and pro-fibrotic mediators such as cytokines, chemokines, and growth factors play a major role. New strategies like stem cell-based treatments and precisely cut tissue slice cultures present exciting opportunities for translational research. The creation of potent antifibrotic treatments, which could revolutionise the treatment of chronic liver and kidney illnesses, requires a thorough grasp of these mechanisms and models.

Although existing experimental models have greatly improved our understanding of fibrotic mechanisms, none can fully capture the complexity of fibrosis as it occurs in humans. Combining different animal models with advanced *in vitro* systems offers a more realistic representation of human disease and can strengthen the translational value of preclinical findings. Future studies should also prioritize the identification of reliable biomarkers, the development of non-invasive diagnostic approaches, and the advancement of personalized treatment strategies to enable earlier intervention and more effective disease management.

In summary, ongoing refinement of experimental tools, together with deeper exploration of molecular and immune-driven pathways, is crucial for successfully translating antifibrotic research into clinical practice. These efforts have the potential to slow or even reverse disease progression, minimize organ failure, and ultimately improve the quality of life for individuals affected by chronic liver and kidney disorders.
